# The relations of job stress dimensions to safety climate and accidents occurrence among the workers

**DOI:** 10.1016/j.heliyon.2021.e08082

**Published:** 2021-09-27

**Authors:** Amir Hossein Khoshakhlagh, Saeid Yazdanirad, Yaser Hatamnejad, Elham Khatooni, Sohag Kabir, Ali Tajpoor

**Affiliations:** a Department of Occupational Health Engineering, Faculty of Health, Kashan University of Medical Sciences, Kashan, Iran; b Social Determinants of Health (SDH) Research Center, Kashan University of Medical Sciences, Kashan, Iran; c School of Health, Shahrekord University of Medical Sciences, Shahrekord, Iran; d Modeling in Health Research Center, Shahrekord University of Medical Sciences, Shahrekord, Iran; e Students' Scientific Research Center, Tehran University of Medical Sciences, Tehran, Iran; f Department of Epidemiology and Biostatistics, School of Public Health, Tehran University of Medical Sciences, Tehran, Iran; g Department of Computer Science, University of Bradford, BD7 1DP, Bradford, UK; h Department of Occupational Health, Faculty of Medical Sciences, Tarbiat Modares University, Tehran, Iran

**Keywords:** Job stress, Workplace, Safety climate, Accident prevention, Regression analysis

## Abstract

Based on a literature review, likely, there is a relationship between job stress and safety climate, and in this way, the accident occurrence is affected. Therefore, the present study was aimed to investigate the relations of job stress dimensions to safety climate and accidents occurrence among the workers using regression models. This cross-sectional study was carried out on 1530 male employees in 2019. People were randomly selected from various departments. The participants filled out the questionnaires, including demographical information and accident history questionnaire, the NIOSH generic job stress questionnaire, and the Nordic safety climate questionnaire. In addition, information on occupational experience and accident history was obtained from the health unit of the petrochemical company. In the end, data were analyzed using statistical tests of bivariate correlation, multivariate correlation, and logistic regression. Based on the bivariate analysis, the variables of job satisfaction (0.998), problem at work (0.900), depression (-0.836), and physical environment (-0.796) among the job stress dimensions had the highest correlation coefficients with the total score of the safety climate, respectively. The results of the logistic regression analysis with the adjustment of the effect of the safety climate indicated that the relationships between the dimensions of the job satisfaction (Wald = 6.50, OR = 4.96, and p-value<0.05) and social supports (Wald = 5.88, OR = 3.20, and p-value<0.05) with the accident occurrence were significant. To increase the positive safety climate and decrease the accident occurrence, industries must try to reduce job stress in the workplaces through controlling the important factors, such as low job satisfaction and poor social supports.

## Highlight

• Job satisfaction had the highest correlation coefficient with the safety climate.
• Management's safety justice possessed the greatest correlation coefficient with the job stress.
• There were significant relationships between job satisfaction and social supports with accident occurrence.

## 1 Introduction

Nowadays, occupational accidents are considered as one of the important and serious potential sources of threats to human health, economy, society, and environment (Comberti et al., 2018). The third rank of the cause of human mortality in the world and the second rank in Iran belong to occupational accidents and injuries (Izadi et al., 2019). In addition, the international labor organization (ILO) estimates the cost of occupational accidents and work-related diseases as four percent of the gross national product (GNP) (Yilmaz and Ҫelebi, 2015). These accidents have various contributing factors. Hollnagel et al. have introduced two types of approaches, including safety I and safety II, to investigate and decrease job accidents. Safety I, as a reactive approach, studies what that goes wrong. While safety II, as a proactive approach, focused on what that goes right. One of the measures for safety II is the creation of positive organizational conditions, which can affect personal behavior and control accidents (Hollnagel, 2018). This condition can be provided through different agents such as decreased job stress and increased positive safety climate. Of course, the correct paths should be investigated to implement effective measures.

Work-related stress is the substantial imbalance between a person's capabilities and job demands to cause great consequences (De Jonge and Dormann, 2017). Based on the reports of the World health organization (WHO), more than half of the employees in the developed countries suffer from occupational stress (Torshizi and Ahmadi, 2011). In general, stress is divided into two types, including physical stress and psychological stress (De Jonge and Dormann, 2017). Physical stress can stimulate the biological responses in reaction to a stressful situation via releasing the hormones and occasion the effects such as sleep disorders, headache, and skin problems (Leung et al., 2012). On the other hand, psychological stress shows an intensive trauma experience, which can cause the effects such as anxiety, sadness, anger, and tension at the workplace (Luan et al., 2020). Various occupational dimensions, such as job control, conflict at work, job satisfaction, mental demand, physical environment, social support, workload, and responsibility can contribute to job stress in the workplace (Kazronian et al., 2013). Several studies have been demonstrated the effect of job stress on accidents. Goldenhar et al. concluded that occupational stressors play an important role in 37 percent of industrial accidents and injuries (Goldenhar et al., 2003). Kim et al. also observed that high job stress was associated with the occurrence of occupational injury among firefighters (Kim et al., 2016). Leung et al. represented a model based on which the job stress impresses on accidents through safety behavior (Leung et al., 2016). The results of the studies show that job stress plays a significant role in the occurrence of unsafe behaviors and accidents by decreasing concentration, distraction, memory impairment, work hesitation, and decision-making power (Day et al., 2012). Moreover, job stress can affect accident occurrences through other paths. One of the probable paths is the changed safety climate. This factor shows the employees' perceptions of the organizational prioritization to the workplace's safety issues (Zohar, 2000). Safety climate has various dimensions such as management's safety priority, commitment, and competence, management's safety empowerment, management's safety justice, workers' safety commitment, and workers' safety priority and risk non-acceptance (Casey et al., 2017). The results of a study performed by Ajslev et al. showed that the safety climate has a reverse relationship to the accident occurrence (Ajslev et al., 2017). On the other hand, Kuo concluded that the job stressors impress on the occupational commitment of police officers (Kuo, 2015). Haque and Aston also investigated the relationship between occupational stress and organizational commitment. The results showed that stress influences the employees' performance and organizational commitment so that low occupational stress and high social support at the workplace can increase it (Haque and Aston, 2016). Therefore, likely, there is a relationship between job stress and safety climate, and in this way, the accident occurrence is affected. The identification of the substantial dimensions and relationships helps to plan the measures for reducing accidents. However, to the best of the knowledge of the authors, no studies have examined them. Therefore, the present study was aimed to investigate the relations of job stress dimensions to safety climate and accidents occurrence among the workers using regression models.

## 2 Material and methods

**Figure 1:**
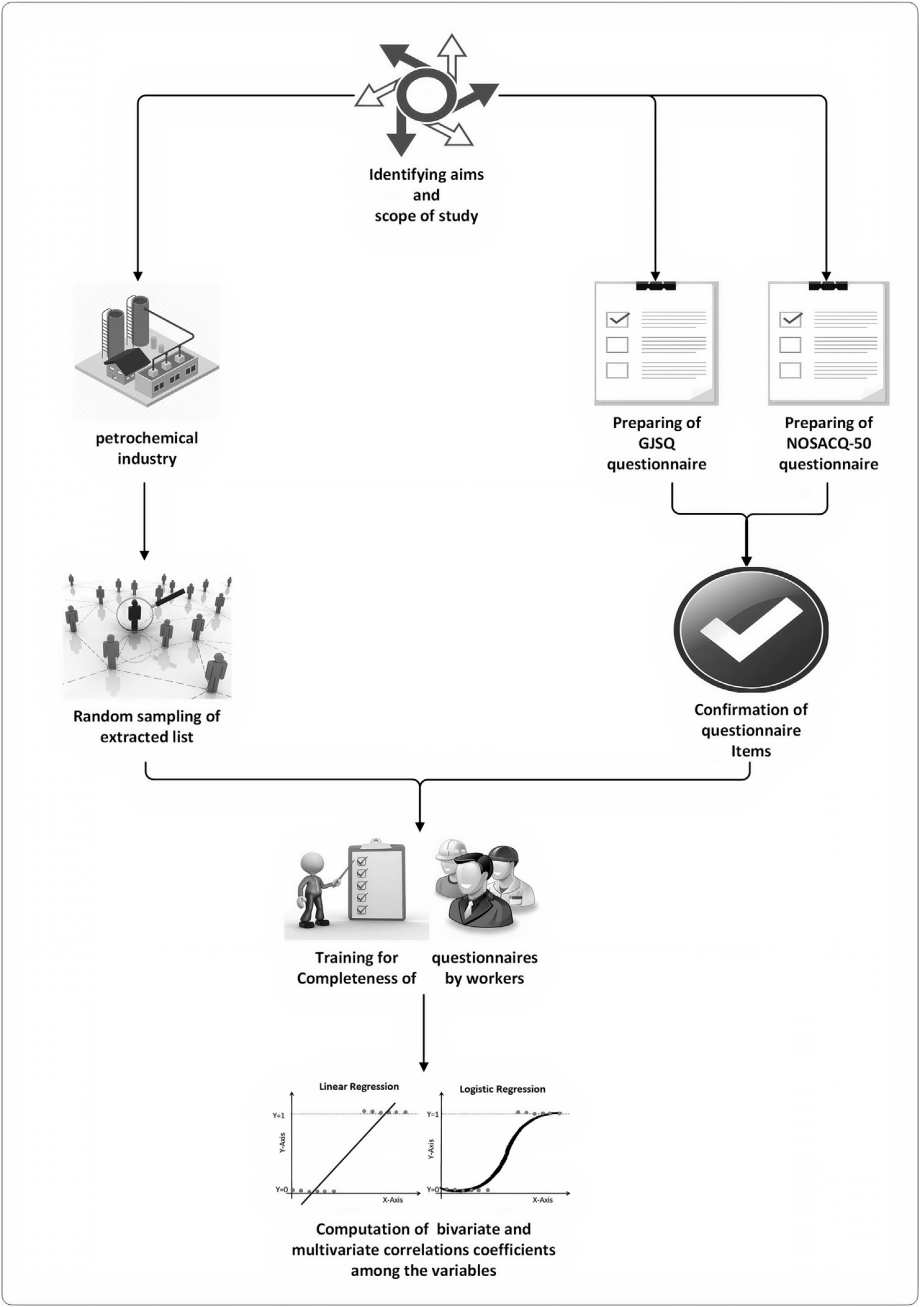
The workflow of the method applied in this study.

Figure 1 shows the workflow of the method applied in this study.

### 2.1 Participants

To investigate the stated assumption, a cross-sectional study was carried out in the summer of 2020 in Asaluyeh petrochemical company in Iran. At the first phase, a list of people occupied in various departments, including 4621 persons, was prepared, and 2100 male workers (45.44%) were randomly selected. Given the low numbers of female employees in this company, they were not entered into the study. Also, it should be noted that most of the workers in industrial environments from Iran are males, and females are mostly employed in administrative jobs. In the next step, the medical records of these workers were studied and those with the inclusion and exclusion criteria were invited into the study. Finally, 1742 people (33.11%) remained in the study. Of them, 1530 persons (87.83%) completed the questionnaires. Inclusion criteria comprised having work experience higher than one year and having literacy. Exclusion criteria included the lack of cooperation and the lack of enough attention to complete the questionnaires. People from different departments of the petrochemical industry, including technical, electrical, machinery, maintenance, mechanical, welding, turning, and supervision parts participated in the study. The number of chosen people in each department was determined based on the ratio of the number of employees in each department to the total number of them. The lowest and highest relative frequencies belonged to supervisors and repairing workers with 3.6% and 18.4%, respectively. The protocol for performing this study was reviewed and approved by the medical ethics committee of Tehran University of medical sciences. All steps of the study were in accordance with the ethical code IR.TUMS.VCR.REC.1398.558. All participants confirmed consciously the consent form provided by the committee.

### 2.2 Data acquisition

Before the onset of the study, the aims and steps of the research were clarified to the individuals. Then, they were trained to complete the questionnaires. The participants filled out the questionnaires in the presence of the researchers in their free time. The questionnaires included demographical information and accident history questionnaire, the NIOSH generic job stress questionnaire (GJSQ), and the Nordic safety climate questionnaire (NOSACQ-50). In addition, information on occupational experience and accident history was obtained from the health unit of the petrochemical company.

### 2.3 Data collection instruments

#### 2.3.1 Demographical data and accident history questionnaire

The demographic information including age, education, marital status, job type, work department, work experience, body mass index, and smoking habit were received using a researcher-made questionnaire. Moreover, the individuals were asked to state their occupational accident experience and its type in the past year.

#### 2.3.2 NIOSH generic job stress questionnaire (GJSQ)

The NIOSH generic job stress questionnaire (GJSQ) designed by the US national institute for occupational safety and health (NIOSH) was used to evaluate the various job stress items among subjects. So far, this questionnaire has been applied in several studies with different purposes. For example, Sugawara et al. evaluated the occupational stress among mental health nurses using GJSQ (Sugawara et al., 2017). Inoue et al. estimated some job stress dimensions of Japanese employees occupied in hospitals and medical facilities, transportation, manufacturing, and the information technology, pharmaceutical, and service industries using this questionnaire (Inoue et al., 2014). In the present study, GJSQ was used to assess the job stress dimensions among the workers of a petrochemical company. The NIOSH generic job stress questionnaire (GJSQ) comprised 21 dimensions, including background information (7 items), conflict at work (16 items), job control (16 items), employment opportunities (4 items), somatic complaints (17 items), general job information (12 items), health condition (24 items), self-esteem (10 items), job requirements (10 items), job satisfaction (4 items), mental demands (5 items), non-work activities (7 items), depression (20 items), physical environment (10 items), problems at work (6 items), social support (12 items), work hazards (5 items), work limitations (5 items), workload and responsibility (11 items), role conflict and ambiguity (14 items), and job future ambiguity (5 items). Kazronian et al. (2013) translated this questionnaire to the Persian version and validated it among Iranian firefighters. Any of the questions and dimensions were removed and changed in this version. They resulted that Cronbach's alpha coefficient of this questionnaire was greater than 0.70 and its intra-cluster correlation coefficient was equal to 0.70 (Cassidy, 1999). However, in the present study, Cronbach's alpha coefficient of the questionnaire and coefficient of intra-class correlation was computed again. The options for answers were different, including yes or no, false and true, Likert from one to five, Likert from one to three, and open-ended replies (ÇETİNKAYA & Dicle, 2017). However, the scores of all dimensions were normalized between values of one and five. The dimensions with the qualitative and non-Likert answers including background information, general job information, health condition, non-work activities, and work limitations were removed. The total score of each of remained dimensions was also obtained by calculating the mean value of the scores of its questions.

#### 2.3.3 Nordic safety climate questionnaire (NOSACQ-50)

It is a valid instrument evaluating the safety climate. A team of experts from several Nordic countries including Denmark, Norway, Iceland, Finland, and Sweden designed it in 2011 (Kines et al., 2011). So far, this questionnaire has been used in several studies with different purposes. For example, Fargnoli and Lombardi assessed the safety climate in agricultural activities using the NOSACQ-50 (Fargnoli and Lombardi, 2020). Marin et al. also applied this questionnaire for evaluating the perceptions of safety climate across construction personnel (Marin et al., 2019). In the present study, the NOSACQ-50 was exploited to estimate the safety climate dimensions among the workers of a petrochemical company. The tool contains fifty items and seven dimensions, including management's safety priority, commitment, and competence (9 items), management's safety empowerment (7 items), management's safety justice (6 items), workers' safety commitment (6 items), workers' safety priority and risk non-acceptance (7 items), safety communication, learning, and trust in co-workers’ safety competence (8 items), and workers' trust in the efficacy of safety systems (7 items) (Kines et al., 2011). In 2016, Yousefi et al. translated this questionnaire to the Persian version and evaluated its validity and reliability in Iran. Any of the questions and dimensions were omitted and altered in this version (Yousefi et al., 2016). Cronbach's alpha coefficient was calculated to be 0.94. However, in the present study, Cronbach's alpha coefficient of the questionnaire and coefficient of intra-class correlation was computed again. In this questionnaire, the subjects answer the questions using a Likert scale from one to four, including strongly disagree, disagree, agree, and strongly agree. The mean value of the scores related to the questions of each dimension was considered as the total score of it.

### 2.4 Data analysis

Data were entered into the SPSS software version 24. At first, descriptive statistics were computed. Then, the expectation maximization method was used to calculate and replace the missing values. Cronbach's alpha coefficient and internal correlation coefficient resulted from two-way mixed variance analysis were also applied to evaluate the reliability of the questionnaires. In addition, the bivariate and multivariate correlations coefficients among the dimensions of the job stress and safety climate were calculated. Furthermore, binary logistic regression analysis was used to investigate the effect of the job stress dimensions on the accident occurrence with the adjustment of the total score of safety climate. The people in terms of accident occurrence were divided into two groups, including with and without occupational accident experience. None of the variables was omitted, and all of them are included in the model. Hosmer and Lemeshow's goodness of fit test was applied to evaluate the adequacy of the model. The significant level was considered as 0.05.

## 3 Results

Based on the results, Cronbach's alpha coefficients of all dimensions of the Nordic safety climate questionnaire were calculated as values greater than 0.90. Additionally, results of the two-way mixed model showed that the values of intra-class correlation coefficients of ICC1 and ICC2 related to the dimensions of this questionnaire were higher than 0.565 and 0.912, respectively. Also, the results revealed that all dimensions of the NIOSH generic job stress questionnaire had Cronbach's alpha coefficients greater than 0.80. Moreover, the intra-class correlation coefficients of ICC1 and ICC2 related to the dimensions of this questionnaire were calculated by values higher than 0.546 and 0.828, respectively.

**Figure 2:**
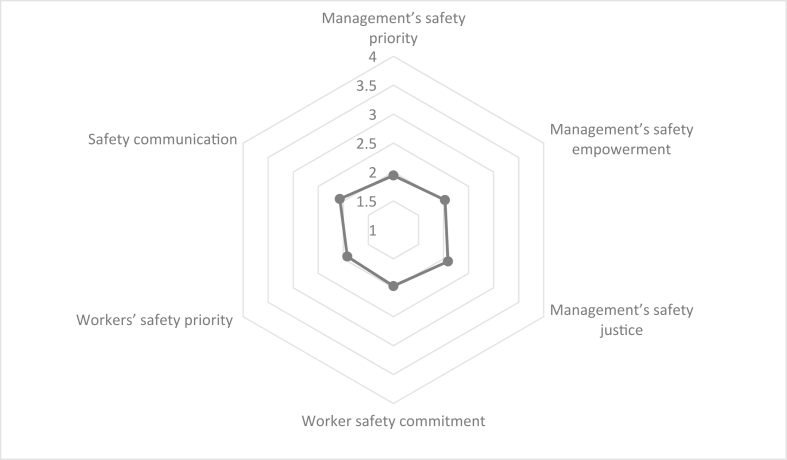
Mean values of scores related to the safety climate dimensions.

**Figure 3:**
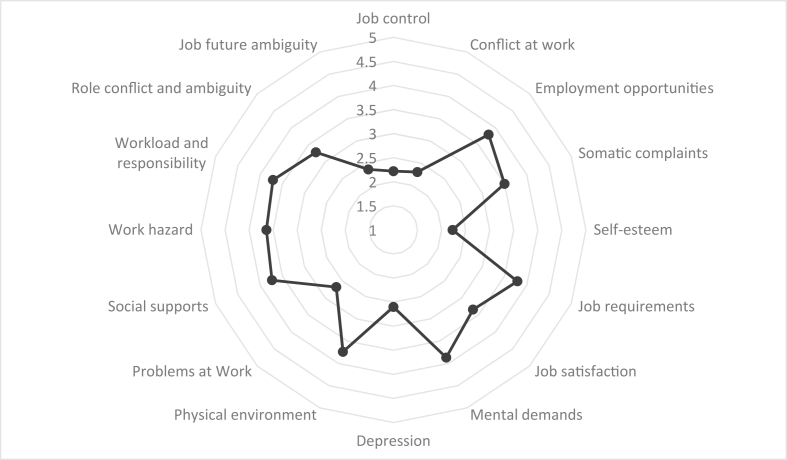
Mean values of scores related to the job stress dimensions.

Table 1 presents descriptive statistics of the demographic variables of the participants. Based on the results, most participants had an age between 30 to 39 years (50.7 %), education level of diploma (44.9 %), work experience between 5 to 10 years (47.3%), body mass index greater than 25 (54.8 %), repairing and machinery job (36.2 %), marriage history (84.4 %), and smoking experience (58.2 %). Moreover, Table 2 reports the descriptive statistics of the safety climate and job stress dimensions. Figures 2 and 3 also show the mean values of scores related to the dimensions of safety climate and job stress. The results indicated that, among safety climate dimensions, the highest mean scores were related to the variables of management's safety justice (2.09), safety communication, learning, and trust in co-workers’ safety competence (2.07), and management's safety empowerment (2.03), respectively. Of the job stress dimensions, the variables of mental demands (3.87), employment opportunities (3.80), job requirements (3.79), physical environment (3.74), and social support (3.73) also possessed the greatest mean scores, respectively.

**Table 1:** Descriptive statistics related to demographic variables.

Demographic variables		Frequency	Valid percent
Age (years)	20–29	226	14.77
	30–39	775	50.65
	40–49	471	30.78
	50–59	58	3.79
Education degree	Under diploma	247	16.14
	Diploma	687	44.90
	Associate degree	460	30.07
	Bachelor degree	127	8.30
	Master degree	9	0.59
Work experience (years)	1–5	124	8.10
	5–10	724	47.32
	10–15	427	27.91
	15 and higher	255	16.67
Body mass index	17.5–20	105	6.86
	20–25	585	38.24
	25 and higher	837	54.71
Type of job	Technical worker	150	9.80
	Electrical worker	131	8.56
	Machinery worker	273	17.84
	Repairing worker	281	18.37
	Conversion worker	261	17.06
	Turnery worker	187	12.22
	Welding worker	128	8.37
	Mechanic worker	64	4.18
	Supervisor	55	3.59
Marital status	single	238	15.56
	married	1292	84.44
Smoking	yes	891	58.24
	no	639	41.76

**Table 2:** The descriptive information of the safety climate and job stress dimensions.

variable	Dimension	Mean	Std. Deviation	Skewness		Kurtosis	
		Statistic	Statistic	Statistic	Std. Error	Statistic	Std. Error
Safety climate	Management's safety priority, commitment, and competence	1.94	0.78	1.08	0.06	-0.25	0.13
	Management's safety empowerment	2.03	0.71	1.09	0.06	-0.14	0.13
	Management's safety justice	2.09	0.67	1.23	0.06	0.05	0.13
	Workers' safety commitment	1.97	0.79	1.06	0.06	-0.21	0.13
	Workers' safety priority and risk non-acceptance	1.92	0.77	1.08	0.06	-0.18	0.13
	Safety communication, learning, and trust in co-workers’ safety competence	2.07	0.68	1.10	0.06	-0.14	0.13
	Workers' trust in the efficacy of safety systems	1.97	0.78	1.00	0.06	-0.31	0.13
Job stress	Job control	2.22	1.07	1.28	0.06	-0.12	0.13
	Conflict at work	2.30	1.07	1.28	0.06	-0.14	0.13
	Employment opportunities	3.80	1.10	-1.16	0.06	-0.05	0.13
	Somatic complaints	3.50	1.04	-1.17	0.06	-0.08	0.13
	Self-esteem	2.23	1.06	1.19	0.06	-0.17	0.13
	Job requirements	3.79	1.12	-1.31	0.06	0.11	0.13
	Job satisfaction	3.34	0.97	0.87	0.06	-0.41	0.13
	Mental demands	3.87	1.12	-1.09	0.06	-0.24	0.13
	Depression	2.60	1.00	-0.97	0.06	0.06	0.13
	Physical environment	3.74	1.38	-1.27	0.06	-0.18	0.13
	Problems at work	2.68	0.56	0.42	0.06	-0.47	0.13
	Social support	3.73	0.97	-1.16	0.06	-0.04	0.13
	Work hazard	3.64	1.12	-1.22	0.06	-0.05	0.13
	Workload and responsibility	3.71	1.03	-1.32	0.06	0.03	0.13
	Role conflict and ambiguity	3.28	0.94	-1.25	0.06	-0.10	0.13
	Job future ambiguity	2.36	1.06	1.25	0.06	0.06	0.13

**Figure 4:**
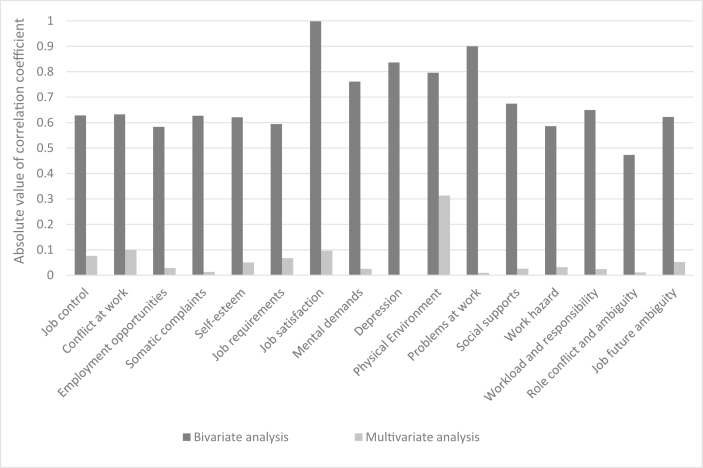
The absolute values of correlation coefficients between the total score of the safety climate and the scores of job stress dimensions.

Table 3 represents the bivariate and multivariate correlation coefficients between the total score of the safety climate and the scores of job stress dimensions. Figure 4 also displays the absolute values of these coefficients. Based on the results of bivariate analysis, all dimensions of job stress had significant relationships with the total score of the safety climate. There were positive relationships between the total score of safety climate and the dimensions of job control, conflict at work, self-esteem, job satisfaction, problem at work, and job future ambiguity scales and the negative relationships between that and the variables of the employment opportunities, somatic complaints, job requirements, mental demands, depression, physical environment, social supports, work hazard, workload and responsibility, and role conflict and ambiguity. The highest correlation coefficients belonged to the dimensions of job satisfaction (0.998), problem at work (0.900), depression (- 0.836), and physical environment (- 0.796), respectively. The results of the multivariate analysis also revealed that the relationships between the total score of safety climate and the dimensions of job control, conflict at work, employment opportunities, self-esteem, job requirements, job satisfaction, physical environment, work hazard, and job future ambiguity were meaningful. The greatest correlation coefficients were related to the dimensions of the physical environment (−0.313), conflict at work (0.099), and job satisfaction (0.096), respectively.

**Table 3:** The bivariate and multivariate correlation coefficients between the total score of the safety climate and the scores of job stress dimensions.

Job stress dimensions	Bivariate analysis			Multivariate analysis		
	Coefficients	95% CI	P value	Coefficients	95% CI	P value
Job control	0.628	0.618 to 0.639	<0.001	0.076	0.034 to 0.117	<0.001
Conflict at work	0.632	0.621 to 0.642	<0.001	0.099	0.056 to 0.142	<0.001
Employment opportunities	- 0.583	- 0.597 to - 0.569	<0.001	- 0.028	- 0.052 to - 0.005	0.018
Somatic complaints	- 0.627	- 0.641 to - 0.613	<0.001	- 0.013	- 0.043 to 0.018	0.410
Self-esteem	0.621	0.609 to 0.634	<0.001	0.050	0.021 to 0.080	0.001
Job requirements	- 0.594	- 0.605 to - 0.583	<0.001	- 0.067	- 0.098 to - 0.036	<0.001
Job satisfaction	0.998	0.971 to 1.024	<0.001	0.096	0.057 to 0.134	<0.001
Mental demands	- 0.761	- 0.779 to - 0.743	<0.001	- 0.025	- 0.057 to 0.006	0.118
Depression	- 0.836	- 0.859 to - 0.814	<0.001	- 0.002	- 0.038 to 0.034	0.919
Physical environment	- 0.796	- 1.826 to - 1.766	<0.001	- 0.313	- 0.418 to -0.208	<0.001
Problems at work	0.900	0.855 to 0.945	<0.001	- 0.009	- 0.034 to 0.016	0.463
Social supports	- 0.674	- 0.688 to - 0.659	<0.001	- 0.026	- 0.058 to 0.007	0.120
Work hazard	- 0.586	- 0.598 to - 0.574	<0.001	- 0.032	- 0.061 to - 0.004	0.025
Workload and responsibility	- 0.649	- 0.661 to - 0.637	<0.001	- 0.024	- 0.062 to 0.014	0.221
Role conflict and ambiguity	- 0.473	- 0.482 to - 0.464	<0.001	- 0.011	- 0.037 to 0.016	0.430
Job future ambiguity	0.622	0.610 to 0.635	<0.001	0.052	0.021 to 0.083	0.001

**Figure 5:**
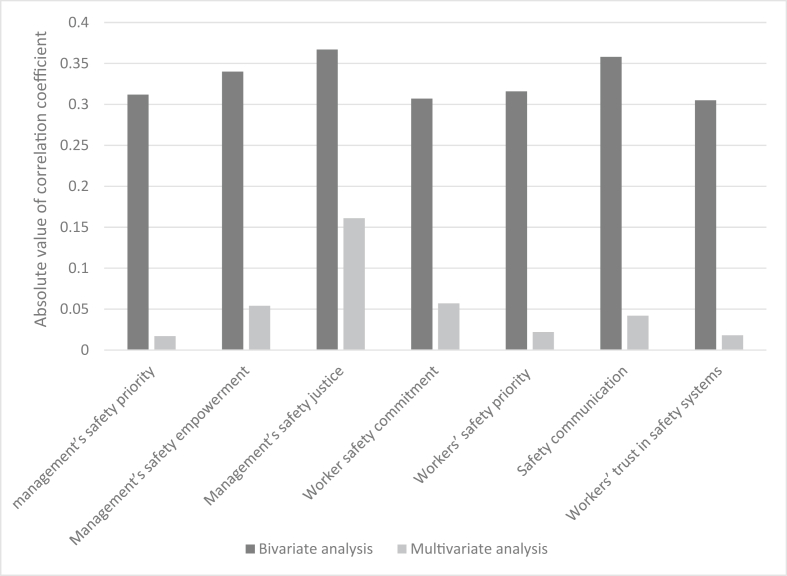
The absolute values of correlation coefficients between the total score of the job stress and the scores of safety climate dimensions.

Table 4 reports the bivariate and multivariate correlation coefficients between the total score of the job stress and the dimensions of the safety climate. Figure 5 also exhibits the absolute values of these coefficients. The bivariate analysis showed that there were significant negative correlations between the total score of job stress and all dimensions of the safety climate. The dimensions of the management's safety justice (- 0.367) and safety communication, learning, and trust in co-workers’ safety competence (- 0.358) had the highest correlation coefficients, respectively. Based on the results of the multivariate analysis, the total score of the job stress showed the significant relationships with dimensions of the management's safety empowerment, management's safety justice, worker's safety commitment, workers' safety priority and risk non-acceptance, and safety communication, learning, and trust in co-workers’ safety competence. The greatest correlation coefficients were related to the dimensions of management's safety justice (- 0.161) and workers' safety commitment (- 0.057), respectively.

**Table 4:** The bivariate and multivariate correlation coefficients between the total score of the job stress and the scores of safety climate dimensions.

Safety climate subscales	Bivariate analysis			Multivariate analysis		
	Coefficients	95% CI	P value	Coefficients	95% CI	P value
Management's safety priority, commitment, and competence	- 0.312	- 0.320 to - 0.305	<0.001	- 0.017	- 0.042 to 0.008	0.191
Management's safety empowerment	- 0.340	- 0.349 to - 0.332	<0.001	- 0.054	- 0.075 to - 0.032	<0.001
Management's safety justice	- 0.367	- 0.375 to - 0.359	<0.001	- 0.161	- 0.182 to - 0.140	<0.001
Worker safety commitment	- 0.307	- 0.315 to - 0.300	<0.001	- 0.057	- 0.079 to - 0.035	<0.001
Workers' safety priority and risk non-acceptance	- 0.316	- 0.324 to - 0.308	<0.001	- 0.022	- 0.042 to - 0.002	0.033
Safety communication, learning, and trust in co-workers’ safety competence	- 0.358	- 0.367 to - 0.349	<0.001	- 0.042	- 0.065 to - 0.018	<0.001
Workers' trust in the efficacy of safety systems	- 0.305	- 0.314 to - 0.297	<0.001	- 0.018	- 0.036 to 0.001	0.064

Additionally, Table 5 describes the effect of job stress dimensions on accident occurrence with adjustment of the total score of safety climate. Based on Hosmer and Lemeshow's goodness of fit test, the model possessed adequate fit. The results of the logistic regression analysis demonstrated that there were significant relationships between the accident occurrence and the dimensions of the job satisfaction (Wald = 6.50, OR = 4.96, and p-value <0.05) and social supports (Wald = 5.88, OR = 3.20, and p-value <0.05).

**Table 5:** The effect of job stress dimensions on accident occurrence with adjustment of total score of safety climate.

Variables in the equation								
	B	S.E.	Wald	df	Sig.	Exp (B)	95% C.I. for EXP(B)	
							Lower	Upper
Job control	0.303	0.681	0.198	1	0.656	1.354	0.357	5.145
Conflict at work	0.152	0.779	0.038	1	0.846	1.164	0.253	5.362
Employment opportunities	- 0.245	0.380	0.415	1	0.519	0.783	0.371	1.649
Somatic complaints	- 0.353	0.507	0.484	1	0.486	0.703	0.260	1.899
Self-esteem	- 0.605	0.543	1.242	1	0.265	0.546	0.189	1.582
Job requirements	0.151	0.443	0.116	1	0.734	1.162	0.488	2.769
Job satisfaction	1.602	0.628	6.501	1	0.011	4.961	1.448	16.993
Mental demands	- 0.458	0.554	0.683	1	0.409	0.633	0.214	1.873
Depression	0.290	0.549	0.280	1	0.597	1.337	0.456	3.921
Physical environment	- 1.315	1.814	0.525	1	0.469	0.268	0.008	9.401
Problems at work	0.336	0.453	0.551	1	0.458	1.400	0.576	3.404
Social supports	1.162	0.479	5.885	1	0.015	3.197	1.250	8.178
Work hazard	0.157	0.423	0.138	1	0.711	1.170	0.511	2.679
Workload and responsibility	- 0.012	0.568	0.000	1	0.984	0.988	0.325	3.007
Role conflict and ambiguity	0.616	0.428	2.073	1	0.150	1.852	0.800	4.283
Job future ambiguity	- 0.030	0.480	0.004	1	0.951	0.971	0.379	2.489
Constant	16.927	14.443	1.374	1	0.241	22448353.350		

## 4 Discussion

The results showed that the used questionnaires have good validity and the information resulted from them is valuable. In general, the results of the bivariate analysis revealed that all dimensions of job stress possessed significant relationships with the total score of the safety climate. While, based on the multivariate analysis, there were significant relationships between the total score of safety climate and the dimensions of job control, conflict at work, employment opportunities, self-esteem, job requirements, job satisfaction, physical environment, work hazard, and job future ambiguity. In the bivariate analysis, the dimension of job satisfaction had the highest correlation coefficient with the total score of safety climate. Nevertheless, in the multivariate analysis, the greatest correlation coefficient with the total score of safety climate belonged to the dimension of the physical environment. Job satisfaction is one of the most important factors affecting job stress.

Huang et al. concluded that the safety climate perceptions of the employees have meaningful relationships with job satisfaction and engagement, and job satisfaction plays a mediator role between safety climate, employee engagement, and turnover rate (Huang et al., 2016). Moreover, Nielsen et al. studied relationships between risk perception, safety climate, and job satisfaction based on the job demands-resources model. The results showed that a positive safety climate has a relation with high job satisfaction and safety climate has a mediator role between risk perception and job satisfaction (Nielsen et al., 2011). The physical environment was another of the important job stress factors affecting the safety climate. It is clear that the poor physical environment such as workplaces with noise, heat, and vibration induces the perceptions of the low management prioritization to the safety and health issues and change the safety climate. Dejoy et al. concluded that environmental conditions could significantly affect the perceived safety climate of employees. The results are consistent with the results of the present study (DeJoy et al., 2004).

Indeed, job dissatisfaction, poor physical environment, and other inappropriate job stress factors can affect organizational justice, as one of the most substantial dimensions of the safety climate. Bakhshi et al. showed that the organizational justice perceptions predict job satisfaction and organization commitment (Bakhshi et al., 2009). The results of the present study also showed that there are significant negative correlations between all dimensions of the safety climate with the total score of the safety climate. However, the highest bivariate and multivariate correlation coefficients between the total score of the job stress with the dimensions of the safety climate were related to the management's safety justice. In a model represented by Fujoshiro and Heaney, there are two-ways relationships between justice and job stress. In this model, work organization and supervisor coworker behaviors influence appraisals of justice and stress. Moreover, job stress and organizational justice impress on each other and thereby cause health problems (Fujishiro and Heaney, 2009). Gyekye and Haybatollahi concluded that the perceived justice level in an organization affects the safety perception and other organizational factors. The social exchange theory adjusts this relationship (Ayim Gyekye and Haybatollahi, 2014). Based on this theory, the response of employees at workplaces depends on their perception of organizational management behaviors (Cropanzano and Mitchell, 2005). Therefore, the lack of job satisfaction and appropriate physical environment makes a bad perception of the organizational behaviors and thereby leads to unsafe acts and accident occurrence. In addition, these factors disorder the balance of the demands and resources. The imbalance of the inputs and outputs causes cognitive failure and a person makes a mistake in performing the job (Day et al., 2012). These errors can also cause accidents.

Based on the results of the present study, when the effect of safety climate was moderated, two factors of job satisfaction and social support were only effective in the occurrence of accidents. These results justify the role of the social exchange theory. Therefore, social supports can compensate the existing failures such as job dissatisfaction. Kula states that both organizational and operational stress has an indirect effect on job satisfaction through supervisor support as a mediator variable (Kula, 2017). Moreover, Woodhead et al. concluded that the support from supervisors and friends or family members as a job resource is associated with lower emotional exhaustion and higher levels of personal accomplishment (Woodhead et al., 2016). Resulted by Havermans et al., social supports including co-worker and supervisor supports can adjust the relationship between psychosocial safety climate and job stress and diminish the negative effects (Havermans et al., 2017). The results of the stated studies and the present study show the important role of job satisfaction and social supports in reducing the negative effects of other stress dimensions. Moreover, the results demonstrated that there is a relationship between job stress scales and safety climate factors. Therefore, the high job stress can create a negative safety climate and vice versa.

One of the limitations of this study was that all participants were male and female workers were not investigated. Other limitations of the present study included the lack of data analysis in various industrial departments and work positions. In addition, the effect of non-occupational stress agents resulted from the family and community environments was not studied and the effect of demographic variables was not considered.

Therefore, it suggests that these limitations be resolved in the next studies.

## 5 Conclusion

In general, the results of the present study showed all dimensions of job stress could be effective on the safety climate. The highest effects belonged to job satisfaction and physical environment. Furthermore, of the safety climate dimensions, management's safety justice showed the greatest correlations with job stress. In addition, with the adjustment of the effect of the safety climate, only two factors of the job stress including job satisfaction and social supports could be effective on the accident occurrence. These results demonstrate the high importance of these factors in accident occurrences. Therefore, the results obtained in this study can be used by the organizations for reducing the accident rate. To increase the positive safety climate and decrease the accident occurrence, industries must try to reduce job stress in the workplaces through controlling the important factors, such as low job satisfaction and poor social supports.

## Declarations

### Author contribution statement

Amir Hossein Khoshakhlagh: Conceived and designed the experiments; Performed the experiments.

Saeid Yazdanirad: Conceived and designed the experiments; Wrote the paper.

Yaser Hatamnejad, Sohag Kabir: Analyzed and interpreted the data.

Elham Khatooni: Contributed reagents, materials, analysis tools or data.

Ali Tajpoor: Performed the experiments.

### Funding statement

This work was supported by 10.13039/501100004484Tehran University of Medical Sciences (97-03-61-38355).

### Data availability statement

Data will be made available on request.

### Declaration of interests statement

The authors declare no conflict of interest.

### Additional information

No additional information is available for this paper.
